# Treatment-Emergent Mania in a Prepubertal Boy

**DOI:** 10.1155/2018/4804912

**Published:** 2018-04-18

**Authors:** Dulangi Maneksha Amerasinghe Dahanayake, Gampolage Swarna Wijetunge

**Affiliations:** Child and Adolescent Mental Health Services, Lady Ridgeway Hospital for Children, Colombo 08, Sri Lanka

## Abstract

Bipolar disorder among children has gained acceptance as a diagnostic entity but continues to pose diagnostic and management challenges due to the developmental differences in children and inadequate evidence for pharmacological management. We present the case of a prepubertal child presenting with an apparent depressive episode who developed treatment-emergent mania when commenced on sertraline. This case highlights the need for further research into the presentations and pharmacological management of prepubertal children with bipolar affective disorder.

## 1. Introduction

A hitherto widely debated concept of bipolar disorder in prepubertal children is now an established diagnostic entity [1]. However, despite several studies in the USA and Europe, there are no published epidemiologic studies on bipolar disorder among children in Asian countries [1]. Furthermore, interpretation of symptoms in the context of a child's developmental age and the higher rates of comorbid disorders remains a challenge, leading to treatment with antidepressants and stimulants [2]. The rates of adverse effects related to such treatment are unclear and pose difficulties in clinical practice [3].

The management of treatment-emergent mania has not been well described and is generally similar to the management of acute manic episodes. The evidence base for the treatment of acute manic episodes in children has expanded over the past few years, but there is still a lack of studies addressing management of bipolar depression and maintenance treatment [4].

The following case report illustrates the clinical presentation and challenges in treating a prepubertal boy with treatment-emergent mania.

## 2. Case Presentation

A 9-year-old boy was brought by his father due to disturbed behaviour with attempts to run in front of traffic over the 2 days preceding the presentation. He was overactive and aggressive, hitting and biting others. His sleep was disturbed.

He has been well until 2 months ago, at which time he refused to go to school citing somatic symptoms. He later reported being physically punished by a school teacher which appeared to have led to the school refusal. During this period, he was irritable and anxious. He reported suicidal ideation and threatened to jump into their well on one occasion, following an argument with his mother. He was socially withdrawn. His sleep was disturbed and he had poor appetite. He was diagnosed as having a depressive episode and commenced on sertraline 25 mg mane 3 weeks prior to the current presentation. His father reported some improvement with this in terms of his activity and dose was increased to 50 mg 2 weeks later. After about 5 days, he became very disturbed, attempting to run off. His father brought him to the hospital as they could not safely contain him at home.

He was the older of 2 siblings and lived with his parents and younger sister. There was a family history of bipolar affective disorder in one of his first cousins. His parents were supportive, but there were marital conflicts and he reported witnessing domestic violence between them. His birth and development history were normal and past medical and psychiatric history were unremarkable. His school performance has been average. Although his parents reported him as being hyperactive and stubborn, his school teachers claimed he was a well-behaved child who could concentrate and completed the tasks he was given. There was evidence of inconsistent parenting styles with his father being authoritarian and mother being very permissive.

On mental state examination, he was an average built boy dressed casually. During the initial interview, he attempted to run off and was restrained physically by his father. He was angry and screamed using obscene words. He was admitted to the in-patient unit. Sertraline was ceased. Clonazepam was used for sedation. As his agitation reduced, he was noted to be socially disinhibited. He had pressure of speech. He did not have flight of ideas. He was extremely irritable. But at times he sang and danced in the ward. There was no suicidal ideation. He expressed self-expansive ideas. He did not have any delusions or hallucinations. He was oriented in time, place, and person. He was distractible and could not engage with cognitive testing. He had poor insight. Physical examination including cardiovascular, respiratory, and nervous systems and the abdomen was unremarkable. He did not have any abnormal movements and bilateral fundi were normal.

Investigations including full blood count, C reactive protein, and thyroid function tests were normal. Electroencephalography recorded in the alert state was normal for the age. Contrast enhanced computerized tomography of the brain was normal.

His presentation met criteria for treatment-emergent mania [3]. He was commenced on risperidone and dose increased to 2 mg twice daily at which point he developed extrapyramidal side effects. As his symptoms persisted beyond one week and there was a positive family history, a diagnosis of bipolar affective disorder, currently manic episode without psychotic symptoms, was made. Sodium valproate was added and dose increased up to 200 mg twice daily. Risperidone was changed to olanzapine after 3 weeks as he continued to have significant emotional and behavioural disturbances; he was extremely irritable and physically aggressive. He showed slow improvement in his mental state after increasing olanzapine to 5 mg twice daily. Following 4 weeks of treatment with olanzapine and sodium valproate, he was discharged home after discussing extensively with his parents regarding the diagnosis, risks, and treatment.

Upon review after 1 week, his parents reported further improvement in his irritability and overactivity and he was continued on the same medication. He was reviewed every 2 weeks for the next month and continued to show improvement in mental state. However, he showed significant weight gain while on treatment. Metabolic screen was done at 4 weeks and investigations were normal. Dietary advice was provided and physical activities were encouraged. Switching to aripiprazole was discussed; however it is not available in the public hospitals in Sri Lanka and parents were not able to afford it. Olanzapine dose was slowly reduced. He then had a relapse of manic symptoms and was commenced on lithium carbonate. Sodium valproate was tailed off. The treating team continued to support parents and liaised with school to ensure smooth transition back to school. His symptoms improved and he was able to go back to school. Olanzapine was tailed off and lithium dose was optimized.

## 3. Discussion

Bipolar affective disorder has been diagnosed in children as young as 5 years [5]. Treatment-emergent mania is described in children and adolescents with the use of antidepressants and stimulant medication [3, 6]. In a retrospective case review, Faedda et al. described patterns of treatment-emergent mania in children with bipolar affective disorder and in 17% of their cases, the first recognition of bipolar illness was in the context of treatment-emergent mania [3]. A higher risk of treatment-emergent mania (76%) was observed with antidepressants (76%) than stimulants (24%) in this case review. The authors further reported that treatment-emergent mania was not different to bipolar disorder in symptoms, course of illness, response to treatment, and the presence of a family history. The case we described met criteria for treatment-emergent mania employed in the above study; the initial presentation was diagnosed as a depressive episode warranting treatment with an antidepressant, which led to an episode of mania within one week of increasing the antidepressant dose. Previous literature described high levels of antidepressant usage in children with bipolar affective disorder, presumably due to the complex and confusing presentations, especially in prepubertal children [7–9]. The differentiation of depressive episodes from mixed mood episodes can be especially difficult in children due to the atypical features seen in childhood depression. In retrospect, it is possible to consider that the case we described might have initially presented with a mixed mood episode rather than a depressive episode, which would have warranted treatment with an atypical antipsychotic rather than an antidepressant. The presence of a family history of bipolar affective disorder should also caution against using an antidepressant, especially as it is one of the most heritable of medical disorders [10].

Management of bipolar disorder in prepubertal children poses challenges due to an inadequate evidence base, especially for bipolar depression and maintenance management, as well as side effect burden. In addition, most medications are not licensed for use in young children.

Studies have shown evidence for second-generation antipsychotics in treating manic episodes in children, including prepubertal children [11, 12]. These have compared second-generation antipsychotics with mood stabilizers in acute manic episodes and shown higher efficacy for the antipsychotics. A trial among 6 to 15 year olds showed higher responses to risperidone [11]. Lithium also has evidence as a treatment for manic episode in children, with a study among 7 to 17 year olds showing superior efficacy for lithium as compared to placebo [13]. Studies on efficacy of divalproex compared to lithium and placebo in acute manic episodes in children have shown mixed results [14, 15]. Nevertheless, there are constraints on practice as only aripiprazole and lithium are licensed for treatment of mania among children in the UK, this being only for children above 12 years [4]. The US Food and Drug Administration has approved risperidone, aripiprazole, and quetiapine for children aged 10 years or above and olanzapine from 13 years as well as lithium for children 12 years or older [16]. The child we described was 9 years old at the time of presentation and the treating team discussed medications, their effects, and side effects with parents in detail, prior to starting pharmacological treatment, as the use was outside licensed indications. Lithium was considered as an add-on therapy early in treatment, but after discussing the side effect profiles and need for regular monitoring with parents, a trial of sodium valproate was commenced. However, he was later switched to lithium carbonate, with which the symptoms improved.

Weight gain was another significant problem encountered in the management of the child described. Studies have shown that younger children are at much higher risk of metabolic side effects of second-generation antipsychotics [17]. The treatment team was vigilant about these effects and closely monitored weight and other metabolic parameters in addition to liaising with the dietician and encouraging physical activities. Olanzapine was tailed off as early as possible, but this proved challenging and the team did not have the option of trialing an alternative second-generation antipsychotic with low metabolic risks as these are not available free of charge in the public hospitals and the child's family were unable to afford them.

The difficulties encountered in managing a prepubertal boy presenting with mood symptoms highlight the need for further research into childhood bipolar disorder and its management. The challenges lie in the lack of available evidence for pharmacological agents which has led to restricted licensing, especially for children younger than 10 years. Furthermore, practicing in a public hospital setting of a lower middle-income country leads to further barriers as the repertoire of medication available for use is even lower.
